# Refocus on Immunogenic Characteristics of Convalescent COVID-19 Challenged by Prototype SARS-CoV-2

**DOI:** 10.3390/vaccines11010123

**Published:** 2023-01-04

**Authors:** Xiaorong Huang, Chumin Liang, Manman Li, Huimin Chen, Zhaowan Li, Qianqian Ruan, Ximing Hu, Lilian Zeng, Huifang Lin, Wei Zhao, Jianpeng Xiao, Limei Sun, Jiufeng Sun

**Affiliations:** 1 School of Public Health, Southern Medical University, Guangzhou 510515, China; 2 Guangdong Provincial Institute of Public Health, Guangzhou 510300, China; 3 Guangdong Provincial Center for Disease Control and Prevention, Guangdong Workstation for Emerging Infectious Disease Control and Prevention, Guangzhou 510317, China; 4 School of Mathematics and Computing Science, Guilin University of Electronic Technology, Guilin 541004, China; 5 Department of Public Health and Preventive Medicine, School of Medicine, Jinan University, Guangzhou 510632, China; 6 School of Public Health, Sun Yat-sen University, Guangzhou 510080, China

**Keywords:** COVID-19, Variants of Concern, SARS-CoV-2, IgG, coronavirus, neutralizing antibody

## Abstract

Background: Mass basic and booster immunization programs effectively contained the spread of the Severe Acute Respiratory Syndrome Coronavirus 2 (SARS-CoV-2) virus, also known as COVID-19. However, the emerging Variants of Concern (VOCs) of COVID-19 evade the immune protection of the vaccine and increase the risk of reinfection. Methods: Serum antibodies of 384 COVID-19 cases recovered from SARS-CoV-2 infection were examined. Correlations between clinical symptoms and antibodies against VOCs were analyzed. Result: All 384 cases (aged 43, range 1–90) were from 15 cities of Guangdong, China. The specific IgA, IgG, and IgM antibodies could be detected within 4–6 weeks after infection. A broad cross-reaction between SARS-CoV-2 and Severe Acute Respiratory Syndrome Coronavirus, but not with Middle East Respiratory Syndrome Coronavirus was found. The titers of neutralization antibodies (NAbs) were significantly correlated with IgG (r = 0.667, *p* < 0.001), but showed poor neutralizing effects against VOCs. Age, fever, and hormone therapy were independent risk factors for NAbs titers reduction against VOCs. Conclusion: Humoral immunity antibodies from the original strain of COVID-19 showed weak neutralization effects against VOCs, and decreased neutralizing ability was associated with initial age, fever, and hormone therapy, which hindered the effects of the COVID-19 vaccine developed from the SARS-CoV-2 prototype virus.

## 1. Introduction

Severe Acute Respiratory Syndrome Coronavirus 2, (SARS-CoV-2) is a novel coronavirus also known as COVID-19. It is an easily transmitted, acute respiratory infectious disease, genus Beta Coronavirus [1], which is one of seven coronaviruses identified that can infect humans. These include the COVID-19 strain, -, Severe Acute Respiratory Syndrome Coronavirus (SARS-CoV), Middle East Respiratory Syndrome Coronavirus (MERS-CoV), coronavirus OC43 (HCoV-OC43), coronavirus HKU1 (HCoV-HKU1), coronavirus NL63 (HCoV-NL63), and coronavirus 229E (HCoV-229E) [2,3].

COVID-19 first appeared in China in 2019, and the World Health Organization declared it a worldwide pandemic in early 2020. In the ensuing three years, the pandemic has caused irreversible destruction in global public health and caused great social and economic burdens [2]. As of October 2022, cumulative COVID-19 cases were over 618 million, with 6.5 million deaths [4]. Vaccines were once considered as the key for elimination of the COVID-19 pandemic. However, the continued circulation of the SARS-CoV-2 virus accelerated the development of novel mutations in the virus genome and resulted in a number of Variants of Concern (VOCs), including Alpha in southeast England [5], Beta [6] and Omicron [7] in South Africa, Gamma [8] in Brazil, and Delta [7] Variants in India. Consequently, the immune protection effects of the vaccine that works by activating antibody responses to the virus were lessened in the emerging VOCs, thus increasing the risk of breakthrough infection [9]. In particular, Omicron variants, the most highly infectious VOCs recorded [10], caused more breakthrough infections than any other VOCs due to substantial resistance to available vaccines [11,12,13].

All the current COVID-19 vaccines, e.g., CoronaVac (Sinovac), BBIBP-CorV (Sinopharm BIBP), BNT162b2 (Pfizer-BioNTech), and mRNA-1273 (Moderna), were designed to fight the prototype SARS-CoV-2 strain. There was growing concern that these first generation vaccines would not provide protection against VOCs, and several second generation vaccines which target two or three VOCs, e.g., prototype, Delta, and Omicrons were under development. Nevertheless, the mass basic and booster immunization program with the first generation vaccine provided some degree of protection against severe disease and death [14]. However, the capability of the emerging VOCs to evade the vaccine immune protection and increase the risk of reinfection, meant it was necessary to evaluate immunogenic characteristics of the prototype virus in infected patients. In this study, we introduced the characteristics of humoral antibodies extracted from prototype SARS-CoV-2 infection, evaluated the landscape cross-reactivity among seven human coronaviruses, and assessed the resistance of the COVID-19 convalescent cases serum to Delta, Beta, Omicron BA.1, and Omicron BA.2 VOCs. In addition, we tested the correlations between the reduction of neutralization of the virus and clinical influencing factors.

## 2. Materials and Methods

### 2.1. Patients’ Information

In total, 384 COVID-19 convalescent hospitalized cases (309 moderate, 56 severe, and 19 critical cases) were collected from 21 hospitals in 15 cities of Guangdong province from January to February 2020. All the cases were laboratory-confirmed COVID-19 cases. Clinical data, including case demographics and date of onset, were retrieved from hospital records. The study was approved by the institutional review committee of the Guangdong provincial center for disease control and prevention. The case was defined according to the COVID-19 clinical guidelines for prevention and control (ninth edition).

### 2.2. Specimen Collection and Storage

According to the clinical diagnosis and treatment guidelines, 384 blood specimens were collected from COVID-19 cases. The specimens were collected and stored at 4 °C (Table S1) and transported to the Guangdong provincial center for disease control and prevention under the same conditions. All blood specimens were separated from serum on the day of collection. The evacuated blood were centrifuged at 3000 rpm/min for 5 min (Thermo Fisher Scientific, Dreieich, Germany), and the serum was transferred into new 2 mL cryogenic vials. All serum specimens were then stored at −80 °C and thermally inactivated at 56 °C for 30 min before assay.

### 2.3. Nucleic Acid Extraction and rRT-PCR

The treatment of clinical specimens was as previously described [15]. The total RNA was extracted using the pre-installed viral total NA kit-Flex (Fisher Scientific, Labserv, Cat. no. KFRPF-805296). SARS-CoV-2 RNA was analyzed with the commercial rRT-PCR kit (DaAn Gene, Guangzhou, China. Cat. no. DA0931) targeting the ORF1ab and nucleocapsid (N) genes. Amplification was performed on the Applied Biosystems™ 7500 real-time fluorescent PCR instrument (ThermoFisher Scientific, Waltham, MA, USA). A CT value <40 was considered as a SARS-CoV-2 positive sample.

### 2.4. SARS-CoV-2 Humoral IgA, IgG, and IgM Antibodies Test

IgA, IgG, and IgM against SARS-CoV-2 were detected in serum samples using enzyme-linked immunosorbent assay (ELISA) kits (RayBiotech, Peachtree Corners, GA, USA) (Cat. no., IEQ-CoVS1RBD-IgG, IE-CoVS1RBD-IgA and IE-CoVS1RBD-IgM) according to the manufacturer’s protocol as our previous study [16].

### 2.5. Landscape Assay of Coronavirus by Enzyme-Linked Immunosorbent Assay

The S, N, and RBD proteins of SARS-CoV-2 and the S and N proteins of the other six human coronaviruses were from Sino Biological Inc. (Beijing, China). All proteins were suspended at 1 µg/mL in PBST (SARS-CoV-2 S protein was suspended at 2 µg/mL). The protein suspension was added to each well of the 96-well plate (100 µL/well) and incubated overnight at 4 °C. Then, plate wells were washed three times with 300 µL of PBST each time on the automatic plate washing machine (BioTek 405 TS, Winooski, VT, USA). Nonfat dry milk (10%) prepared in PBST as a blocking solution was added to the plates (200 µL/well) and incubated at 37 °C for 1 h. Plate wells were washed again (under the same conditions as before). The serum specimens diluted in nonfat dry milk prepared in PBST (100 µL/well, serum specimens were heat inactivated for 30 min at 56 °C and diluted at 1:100). The diluted specimens were incubated for 1 h at 37 °C, and then plate wells were washed for three times. The anti-human IgM-HRP or anti-human IgG-HRP was added to the plates (100 µL/well) and incubated at 37 °C for 1 h. Plate wells were washed again. The TMB substrate was added to each well (100 µL/well) and incubated for 15 min, followed by the addition of stopping solution (50 µL/well). The optical density (OD) of each well at 450 nm was measured on the enzyme-labeled instrument (BioTek Epoch, Winooski, VT, USA). The wells without the addition of serum served as a background control.

According to the analysis results of SPSS, all the values were subtracted from the blank value before the next comparison. The median of the negative controls for the same protein and the same antibody type was calculated, and then its standard deviations (SD) value was calculated using the median + 2SD as the cut-off value.

### 2.6. SARS-CoV-2 Micro-Neutralization Assay

Prototype SARS-CoV-2 (GDPCC-nCOV-8) and VOCs (Beta, GDPCC2.00004; Delta, GDPCC2.00096; Omicron BA.1, GDPCC.2.00097; and Omicron BA.2, GDPCC.2.00299) were isolated from confirmed COVID-19 cases in Guangdong at Guangdong Provincial Center for Disease Control and Prevention. Vero-E6 cells were seeded in a 96-well plate at 2 × 10^4^ cells per well. An end-point-dilution microplate neutralization assay was performed to measure the neutralization activity of convalescent serum. Duplicates of each serum were incubated with SARS-CoV-2 at a multiplicity of infection (MOI) of 0.1 in MEM (Gibco, Grand Island, New York, NY, USA) with 7.5% inactivated fetal calf serum (FBS, Gibco, USA) at 37 °C for 1 h. Then, the virus–serum mixture was transferred onto a monolayer of Vero E6 cells and incubated for ~72 h for the prototype virus and ~120 h for VOCs at 37 °C, 5% CO_2_.

### 2.7. Statistical Analysis

The statistical analyses for the authentic virus neutralization assessments were performed using GraphPad Prism for calculation of mean value for each data point. Each specimen was tested in duplicate. The neutralization antibody (NAb) titers of the prototype virus and VOCs were log2-transformed prior to analysis, and compared by *t* and *z* test. The correlation between the NAb titers of the prototype virus and IgA, IgM, and IgG antibodies levels were measured using Spearman’s rank correlation coefficient. For analyzing the factors affecting the NAb titers, we first conducted the univariate variance analyses of all the clinical characteristics to select the independent variables that significantly correlated with the change in the outcome measure. Then, the variables selected from the univariate variance analyses were included in the multivariate linear regression model. The level of statistical significance was set at 0.05.

## 3. Results

### 3.1. Clinical Findings of COVID-19 Convalescent Cases

There were 384 cases included in this study, including 196 male (51.1%) and 188 female cases (48.9%). The median age was 43 years (range: 1–90). There were 58 cases (15.1%) in the service industry or as workers, 147 cases (38.3%) were retirees, 37 cases (9.6%) were students, and the rest (21.9%, 84 of 384) were employed in other jobs. Of all the cases, 4.4% (17 of 384) were mild cases, 76.1% (292 of 384) were moderate cases, and 19.5% (75 of 384) were severe and critical cases. A total of 59 cases (15.4%) were hospitalized within 0–14 days, 174 cases (45.3%) within 15–28 days, 133 cases (34.6%) within 29-42 days, and 18 cases (4.7%) over 42 days. The main clinical manifestations were fever (n = 261, 68.0%), cough (n = 183, 47.7%), fatigue (n = 61, 15.9%), muscle pain (n = 32, 8.3%), dyspnea (n = 28, 7.3%), and diarrhea (n = 22, 5.7%). Table 1 summarizes the general clinical characteristics of cases (Table S1).

### 3.2. Cross-Reactivity between SARS-CoV-2 and Six Other Human Coronaviruses

A total of 381 serum specimens were used for ELISA assay. Three samples were excluded because of insufficient volumes. The results showed that 341 serum specimens (341/381, 89.50%) were positive for SARS-CoV-2-S-IgM, 377 serum specimens (377/381, 98.95%) were positive for SARS-CoV-2-S-IgG, 124 serum specimens (124/381, 32.55%) were positive for SARS-CoV-2-N-IgM, and 364 serum specimens (364/381, 95.54%) were positive for SARS-CoV-2-N-IgG (Table S2).

**Figure 1:**
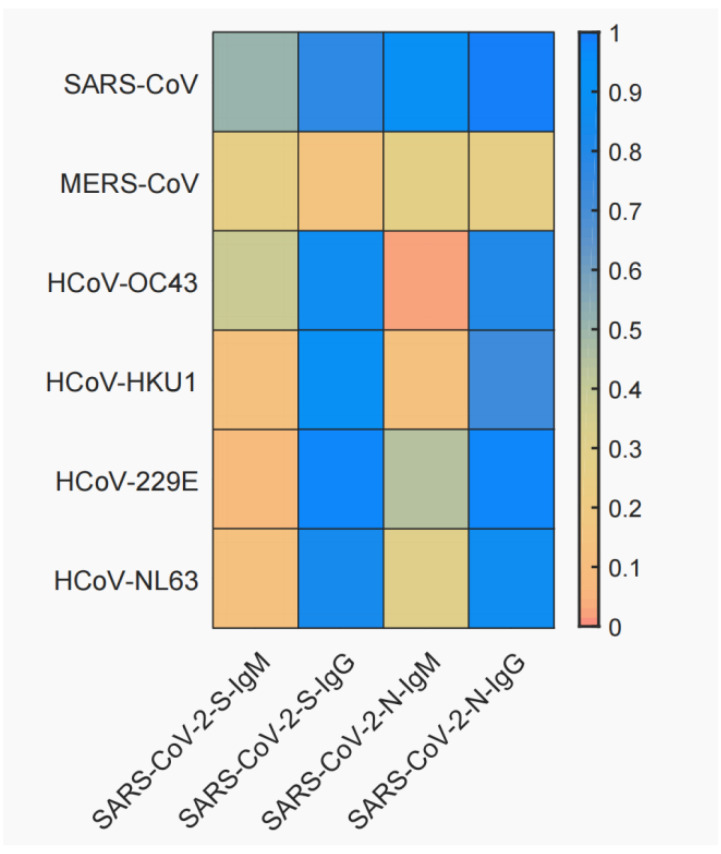
Cross-reactivity of antibodies with six other coronaviruses. The crossover rate was calculated using the actual number of serum specimens involved in the assay as the denominator and the number of positive specimens detected as the numerator.

The cross-reactivity of these positive serum specimens to the other six coronavirus S and N proteins are shown in Figure 1. IgM and IgG reactions of S proteins showed significant cross-reactivity of SARS-CoV-2 with SARS-CoV (S-IgM 51.32%, S-IgG 78.25%, N-IgM 89.52%, N-IgG 100.00%), but not with MERS-CoV (S-IgM 25.59%, S-IgG 14.89%, N-IgM 28.23%, N-IgG 26.18%). In S-IgM, we did not observe obvious cross-reactivity of SARS-CoV-2 with the other four seasonal coronaviruses. On the contrary, in S-IgG, SARS-CoV-2 reacted strongly with HCoV-OC43 (85.79%), HCoV-HKU1 (90.49%), HCoV-229E (97.04%), and HCoV-NL63 (83.83%). The IgM and IgG reaction results of N proteins showed that SARS-CoV-2 cross-reacted more significantly with SARS-CoV, but not with MERS-CoV, which was similar to the results of S proteins. In N-IgM, we observed cross-reactivity of SARS-CoV-2 with HCoV-229E (43.80%), but not with the other three seasonal coronaviruses. On the contrary, in N-IgG, SARS-CoV-2 showed strong cross-reactivity with HCoV-OC43 (80.06%), HCoV-HKU1 (73.03%), HCoV-229E (96.90%), and HCoV-NL63 (87.15%). Overall, it appeared that SARS-CoV-2 showed higher cross-reactivity with SARS-CoV in S-IgG and N-IgG compared with S-IgM and S-IgG, but not for MERS-CoV.

### 3.3. Consistency of NAbs Titers with IgA/IgM/IgG

**Figure 2:**
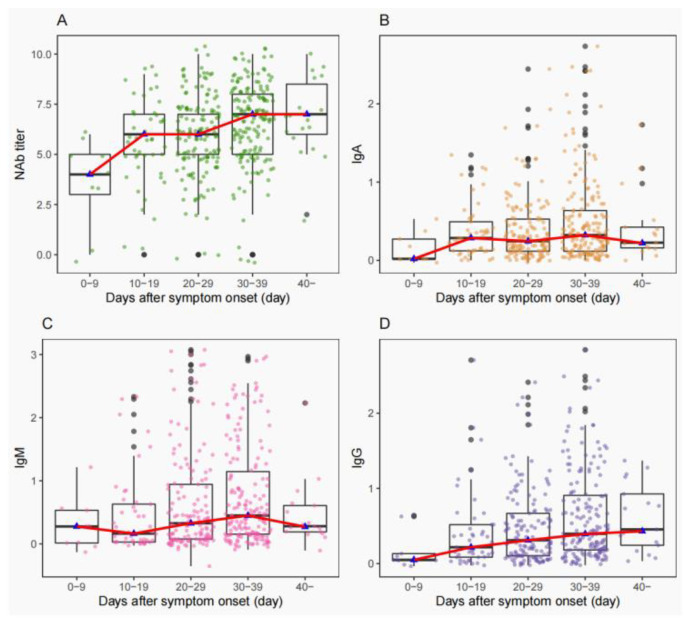
Distribution of titers of neutralization antibody (NAb)/IgA/IgM/IgG with days after symptom onset. The green circles represent the NAb titer, the yellow circles represent the IgA, the red circles represent IgM, and the purple circles represent the IgG. Each circle represents one sample: (**A**) micro-neutralization assay Nab; (**B**) enzyme-linked immunoassay IgA; (**C**) enzyme-linked immunoassay IgM; (**D**) enzyme-linked immunoassay IgG.

To assess the antibody response of cases infected with prototype SARS-CoV-2, we measured NAb levels by micro-neutralization assay and IgA/IgM/IgG antibody levels by ELISA assay. The distributions of NAbs titers and IgA, IgM, or IgG were plotted, and the trends of the curves of NAb titers were correlated with those of IgG (Figure 2A,D). The trends of IgA and IgM curves were similar (Figure 2B,C). The Spearman correlation assay between NAb titers and IgA, IgM, and IgG showed that NAb titers were more correlated with IgG (r = 0.667, *p* < 0.001) than IgA (r = 0.415, *p* < 0.001) and IgM (r = 0.447, *p* < 0.001).

### 3.4. The Decline of NAb Titers of VOCs

**Figure 3:**
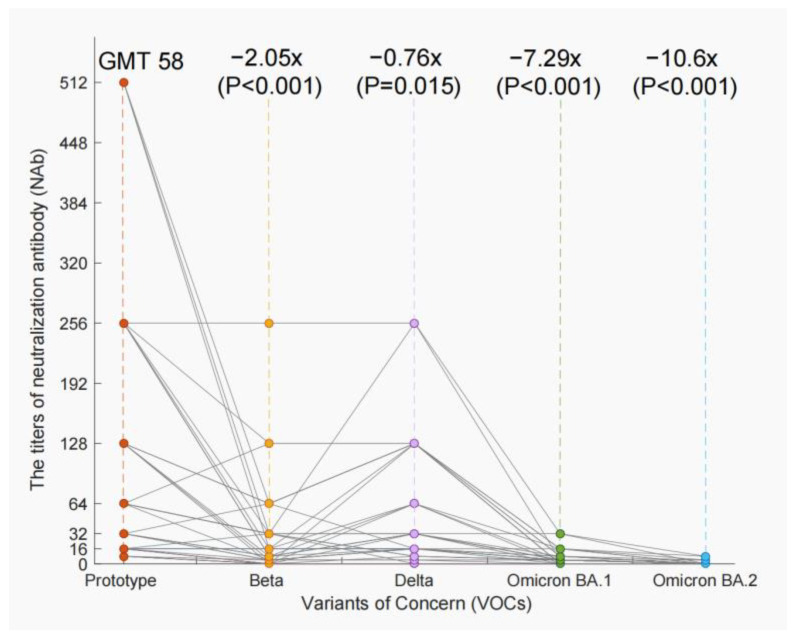
The NAb titers against the prototype virus and a variety of Variants of Concern (VOCs). Neutralization titers against the prototype virus and four VOCs, Beta, Delta, Omicron BA.1, and Omicron BA.2, were tested with the serum of COVID-19 patients. The geometric mean titer (GMT) for the prototype virus was 58, but the VOC Beta decreased to 19 (2.05 fold, *p* < 0.001), Delta decreased to 33 (0.76 fold, *p* = 0.015), Omicron BA.1 decreased to 7 (7.29 fold, *p* < 0.001), and Omicron BA.2 decreased to 5 (10.6 fold, *p* < 0.001).

We evaluated the NAb titers of 36 serum specimens of COVID-19 convalescent cases against the prototype virus and four preventative VOCs. The results showed the NAb titers were significantly decreased against VOCs compared to the prototype virus (Figure 3). The geometric mean titers (GMT) dropped from 58 against the prototype virus to 19 against Beta (2.05 fold, *p* < 0.001), 33 against Delta (0.76 fold, *p* = 0.015), 7 against Omicron BA.1 (7.29 fold, *p* < 0.001), and 5 against Omicron BA.2 (10.6 fold, *p* < 0.001). The most significant decrease of NAb titers was against Omicron VOCs. In addition, we detected negative neutralizing activity in 4 (4/36, 11.11%), 7 (7/36, 19.44%), 11 (11/36, 30.56%), and 31 (31/36, 86.11%) serum specimens when tested with Beta, Delta, Omicron BA.1, and Omicron BA.2 strains.

### 3.5. The Factors Affecting the NAb Titers

The logarithmic of NAb titers was used as the dependent variable, and univariate regression analysis was applied to each variable. The results showed that NAb titers in COVID-19 patients were significantly correlated (*p* < 0.05) with age, occupation, severe disease typing (highest clinical severity), sampling time, fever, whether myalgia was present, mode of oxygenation, whether a noninvasive ventilator was used, whether ICU was required, whether hormone therapy was used, body temperature, neutrophil count, and lymphocyte count (Table S3). Based on the significant correlation factors in the univariate analysis, we further developed a multiple linear regression model to further analyze their correlations. We found that the NAb titers were significantly correlated with age, febrile, and hormone therapy (Table 2).

We further evaluated the factors affecting the decline of NAb titers, and the titers of Beta VOCs were an example. The results showed that the decrease rate of NAb was significantly correlated with age, fever, and hormone therapy (*p* < 0.05) (Table 3), which were the same with factors affecting the NAb titers.

## 4. Conclusions

Antibody profiles of prototype SARS-CoV-2 infection were clarified in many archived studies [17,18,19]. Here, we showed the dynamic antibody profiles of IgA, IgM, and IgG in COVID-19 convalescent patients and found that NAbs were significantly correlated with IgA (r = 0.415, *p* < 0.001), IgM (r = 0.447, *p* < 0.001), and IgG (r = 0.667, *p* < 0.001). In particular, the strongest correlation with IgG indicated that IgG could be used as a substitute marker for NAb production [20].

Then, we tested the landscape cross-reactivity of other six coronaviruses which could cause human infection. The results showed a significant cross-reactivity between SARS-CoV-2 and SARS-CoV, but not with MERS-CoV. Meanwhile, SARS-CoV-2 could cross-react with the other four coronaviruses to variable degrees. Considering that SARS-CoV only temporarily circulated in 2003 to 2004 [21], it is unlikely that most patients were previously infected with SARS-CoV, and the significant cross-reactivity was due to the high sequence homology between SARS-CoV-2 and SARS-CoV (>90%) [19]. No obvious cross-reactivity was observed between SARS-CoV-2 and MERS-CoV, which was consistent with the results derived by Wang et al. [20]. MERS-CoV was predominantly endemic in Middle Eastern countries [22,23]. The genome similarity of SARS-CoV-2 and MERS-CoV was also lower than that with SARS-CoV [24]. Seasonal coronaviruses are common causes of colds, the sero-positive rate within the population is generally high [25,26,27], and there are corresponding antibodies in many individuals [28]. Our results showed that positive serum for SARS-CoV-2 exhibits high seropositivity to a variety of seasonal coronaviruses, suggesting that antibodies to other coronaviruses are present in the serum of most COVID-19 convalescent patients. Other studies also reported that COVID-19 infection leads to increased antibody titers to seasonal coronaviruses [29,30,31]. Regardless, our study confirmed that SARS-CoV-2 has cross-reactive antibodies with other coronaviruses, which was consistent with the conclusion of Woudenberg et al. [32]. It has been observed that the existing immune response against seasonal coronaviruses is protective against SARS-CoV-2 infection [33,34,35], but some studies have shown that this cross-reactivity may exacerbate the severity in COVID-19 patients [36]. Further studies are needed to confirm the effect of seasonal coronavirus infection on SARS-CoV-2 disease progression.

Compared with prototype SARS-CoV-2 strain, serum from COVID-19 convalescent patients showed a significant decrease in neutralization ability against aa variety of VOCs. This finding showed similar trends with the findings of other investigations in the serum of convalescent patients or healthy individuals who had been vaccinated [37,38,39,40,41]. In particular, both basic and booster vaccination with the first generation vaccine showed poor resistance to the present pandemic of Omicron VOCs, which highlight that a second generation vaccine effective against the prototype and VOCs should be implemented as soon as possible [42,43].

Furthermore, we would like to know whether the decrease of NAbs of convalescent COVID-19 patients was associated with any initial clinical symptoms. We found that age, fever, and hormone therapy were the independent risk factors for a dropdown of NAbs. Numerous studies have shown that age [44,45] and fever [46,47] were the risk factors for the development of COVID-19. The clinical therapy for severe COVID-19 using hormones is quite controversial. Some studies showed that glucocorticoids can reduce the mortality of COVID-19 [48], but others found that the efficacy of hormones was not significant and may even bring side effects. Thus, it was not recommended in clinics [49]. Referring to the latest protocol for the diagnosis and treatment of novel coronavirus pneumonia (Ninth Edition), “glucocorticoids” and “interleukin-6 (IL-6) inhibitors” can be used as appropriate for some severe and critical patients [50]. According to the present data, patients with severe COVID-19 were more likely to receive hormone therapy. Unfortunately, the decrease rate of NAbs among these patients was higher than others, which indicated a fast declining tendency of NAbs. Thus, reinfection or breakthrough infections were inevitable for those with higher NAbs when challenged with novel VOCs, e.g., Omicron variants.

In conclusion, we characterized the serum immunogenic profile of prototype SARS-CoV-2 infected COVID-19 patients and indicated a broad cross-reaction with other coronavirus which may hinder the antibody-based clinic diagnosis of COVID-19. The emerging VOCs, e.g., Omicron variants, decreased the immune NAbs evoked from prototype virus infection, and the declining tendency was associated with initial clinical symptoms, including age, fever, and hormone therapy. Given the continuous circulation of novel VOCs, the herd immunity the SARS-CoV-2 vaccine provided was weakening. Nevertheless, booster immunity with the second generation vaccine including prototype and VOCs should be implemented as soon as possible.

## Figures and Tables

**Table 1:** General characteristics of 384 COVID-19 cases (n = 384).

Basic Characteristics		Clinical Characteristics			
	N (%)		N (%)		N (%)
Gender		Fever		Tracheotomy treatment	
Female	188 (48.9)	No	123 (32.0)	No	381 (99.2)
Male	196 (51.1)	Yes	261 (68.0)	Yes	3 (0.8)
Age (years)		Cough		ECMO treatment	
≤39	170 (44.3)	No	201 (52.3)	No	379 (98.7)
40–59	126 (32.8)	Yes	183 (47.7)	Yes	5 (1.3)
≥60	88 (22.9)	Weakness		ICU treatment	
Occupation		No	323 (84.1)	No	350 (91.2)
service	58 (15.1)	Yes	61 (15.9)	Yes	34 (8.8)
retire	147 (38.3)	Dyspnea		CRRT treatment	
worker	58 (15.1)	No	356 (92.7)	No	378 (98.4)
student	37 (9.6)	Yes	28 (7.3)	Yes	6 (1.6)
other	84 (21.9)	Muscle pain		Anti-infective drugs treatment	
Highest clinical severity		No	352 (91.7)	No	167 (43.5)
Mild	17 (4.4)	Yes	32 (8.3)	Yes	217 (56.5)
Moderate	292 (76.1)	Diarrhea		Vasoactive drug treatment	
Severe	75 (19.5)	No	362 (94.3)	No	378 (98.4)
Comorbidity		Yes	22 (5.7)	Yes	6 (1.6)
No	292 (76.0)	Oxygen Inhalation treatment degree		Hormone treatment	
Yes	92 (24.0)	No	130 (33.9)	No	338 (88.0)
Hospital stay (days)		2L	152 (39.6)	Yes	46 (12.0)
0–14	59 (15.4)	3L–4L	57 (14.8)	Temperature(°C)	
15–28	174 (45.3)	5L–6L	6 (1.6)	<37.3	309 (80.5)
29–42	133 (34.6)	High flow	39 (10.2)	≥37.3	75 (19.5)
>42	18 (4.7)	Oxyhydrogen atomizer treatment		Respiration(times/min)	
Aggravation of illness during hospitalization		No	351 (91.4)	≤20	324 (84.4)
No	327 (85.2)	Yes	33 (8.6)	>20	60 (15.6)
Yes	57 (14.8)	Noninvasive ventilator treatment		Pulse(times/min)	
The re-inspection positive		No	342 (89.1)	<60	5 (1.3)
No	270 (70.3)	Yes	42 (10.9)	60–100	329 (85.7)
Yes	114 (29.7)	Tracheal cannula treatment		>100	50 (13.0)
		No	372 (96.9)		
		Yes	372 (3.1)		
Laboratory test characteristics					
	N (%)		N (%)		N (%)
Neutrophil count (×109/L)		Systolic blood pressure (mmHg)		Oxygenation index	
<1.8	23 (6.0)	<90	2 (0.5)	<400	112 (29.2)
1.8–6.3	310 (80.7)	90–139	312 (81.3)	400–500	115 (29.9)
>6.3	36 (9.4)	≥140	58 (15.1)	>500	42 (10.9)
Unknown	15 (3.9)	Unknown	12 (3.1)	Unknown	115 (29.9)
Lymphocyte count (×109/L)		Diastolic blood pressure (mmHg)		Blood oxygen saturation (%)	
<1.0	79 (20.6)	<60	8 (2.1)	<95	11 (2.9)
≥1.0	288 (75.0)	60–89	303 (78.9)	≥95	345 (89.8)
Unknown	17 (4.4)	≥90	60 (15.6)	Unknown	28 (7.3)
Aspartate aminotransferase (U/L)		Unknown	13 (3.4)	White blood cell count (×10^9^/L)	
<40	328 (85.4)	Mean arterial pressure (mmHg)		<4	55 (14.3)
≥40	36 (9.4)	<70	4 (1.0)	4–10	291 (75.8)
Unknown	20 (5.2)	70–105	301 (78.4)	>10	21 (5.5)
Alanine aminotransferase (U/L)		>105	66 (17.2)	Unknown	17 (4.4)
<40	300 (75.1)	Unknown	13 (3.4)		
≥40	64 (16.7)				
Unknown	20 (5.2)				

Data are n (%). For statistical analysis, the Chi-square test and Mann–Whitney U test were used for the comparison of categorical and continuous variables, respectively.

**Table 2:** Multivariate analysis of factors associated with NAb titers.

	Median (log_2_)	β	*p*-Value
Gender			
female	188 (6)	Reference	
male	196 (7)	0.225	0.266
Age			
<40	170 (6)	Reference	
40–59	126 (7)	0.566	0.020
60	88 (7)	0.621	0.041
Highest clinical severity			
Mild	17 (6)	Reference	
Moderate	292 (6)	−0.453	0.360
Severe	75 (8)	0.431	0.438
Fare			
no	123 (5)	Reference	
yes	261 (7)	1.046	<0.001
Comorbidity			
no	292 (6)	Reference	
yes	92 (6)	0.044	0.869
Muscle pain			
No	352 (6)	Reference	
Yes	32 (7)	0.389	0.293
Oxygen inhalation treatment degree			
No	130 (6)	Reference	
2L	152 (6)	0.310	0.212
3L–4L	57 (7)	0.540	0.099
5L–6L	6 (8.5)	1.648	0.059
High flow	39 (7)	0.553	0.209
Noninvasive ventilator treatment			
No	342 (6)	Reference	
Yes	42 (8)	−0.078	0.873
ICU treatment			
No	350 (6)	Reference	
Yes	34 (7)	0.184	0.700
Hormone treatment			
No	338 (6)	Reference	
Yes	46 (8)	0.950	0.024
Temperature(°C)			
<37.3 °C	309 (6)	Reference	
≥37.3	75 (7)	0.474	0.078
Neutrophil count (×10^9^/L)			
<1.8	23 (7)	Reference	
1.8-6.3	310 (6)	−0.036	0.937
≥6.3	36 (7)	0.088	0.877
Unknown	15 (7)	—	
Lymphocyte count (×10^9^/L)			
<1.0	79 (7)	Reference	
≥1.0	288 (6)	−0.181	0.511
Unknown	17 (7)	—	

The values in parentheses are converted from the median by a logarithm based on 2; *p* < 0.05 (two-sided) as statistically significant.

**Table 3:** Multivariate linear regression analysis of factors related to the rate of decline in the NAb titers of Beta.

	Median (log_2_)	β	*p*-Value
Gender			
female	188 (0.600)	Reference	
male	196 (0.667)	0.303	0.136
Age			
<40	170 (0.667)	Reference	
40–59	126 (0.613)	0.692	0.005
60	88 (0.571)	0.661	0.030
Highest clinical severity			
Mild	17 (1.000)	Reference	
Moderate	292 (0.667)	−0.382	0.431
Severe	75 (0.556)	0.583	0.286
Fare			
no	123 (0.667)	Reference	
yes	261 (0.625)	1.003	<0.001
Comorbidity			
no	292 (0.625)	Reference	
yes	92 (0.667)	−0.070	0.795
Muscle pain			
No	352 (0.625)	Reference	
Yes	32 (0.571)	0.293	0.441
Oxygen treatment degree			
No	130 (0.667)	Reference	
2L	152 (0.667)	0.377	0.136
3L–4L	57 (0.571)	0.643	0.052
5L–6L	6 (0.613)	1.594	0.064
High flow	39 (0.571)	0.522	0.240
Noninvasive ventilator treatment			
No	342 (0.625)	Reference	
Yes	42 (0.444)	−0.212	0.664
ICU treatment			
No	350 (0.625)	Reference	
Yes	34 (0.444)	0.068	0.884
Hormone treatment			
No	338 (0.667)	Reference	
Yes	46 (0.500)	0.838	0.044
Temperature(°C)			
<37.3	309 (0.625)	Reference	
≥37.3	75 (0.625)	0.480	0.072
Neutrophil count (×10^9^/L)			
<1.8	23 (0.625)	Reference	
1.8–6.3	310 (0.625)	0.053	0.908
≥6.3	36 (0.667)	0.066	0.907
Unknown	15 (0.667)	—	
Lymphocyte count (×10^9^/L)			
<1.0	79 (0.600)	Reference	
≥1.0	288 (0.625)	−0.357	0.198
Unknown	17 (0.667)	—	

## Data Availability

Not applicable.

## Supplementary Materials

The following supporting information can be downloaded at: https://www.mdpi.com/article/10.3390/vaccines11010123/s1, Table S1: Statistical information of demographic, epidemiological and clinical trial indicators of participants; Table S2: Cross-reactivity between SARS-CoV-2 and six other human coronaviruses. Table S3: Univariate variance analyses of all the general characteristics associated with antibody levels.
