# Supplementary material for: Refocus on Immunogenic Characteristics of Convalescent COVID-19 Challenged by Prototype SARS-CoV-2
Source: Vaccines (Basel). 2023 Jan 4;11(1):123. doi: 10.3390/vaccines11010123 (PMC9866260; doi:10.3390/vaccines11010123)
Supplement: Supplementary file 1 [file vaccines-11-00123-s001.zip › Supplementary Materials-tables s2-s3.pdf]

Supplementary Materials

Table S2. Cross-reactivity between SARS-CoV-2 and six other human coronaviruses.

	SARS-CoV-2-S-IgM	SARS-CoV-2-S-IgG	SARS-CoV-2-N-IgM	SARS-CoV-2-N-IgG
SARS-CoV	51.32%	78.25%	89.52%	100.00%
MERS-CoV	25.59%	14.89%	28.23%	26.18%
HCoV-OC43	38.64%	85.79%	3.31%	80.06%
HCoV-HKU1	13.69%	90.49%	14.05%	73.03%
HCoV-229E	8.31%	97.04%	43.80%	96.90%
HCoV-NL63	13.06%	83.83%	30.33%	87.15%

Table S3. Univariate variance analyses of all the general characteristics associated with antibody levels.

（一）Basic characteristics

Variables	F-value	P-value
Gender	3.403	0.066
**Age (years)**	**3.195**	**0.002**
**Occupation**	**2.535**	**0.040**
**Highest clinical Severity**	**13.962**	**＜0.001**
Comorbidity	2.852	0.092

（二）Clinical characteristics

Variables	F-value	P-value
**Sampling time**	**9.361**	**＜0.001**
**Fever**	**29.287**	**＜0.001**
Cough	0.073	0.787
Weakness	2.808	0.095
**Muscle pain**	**5.976**	**0.015**
Diarrhea	0.004	0.951
Dyspnea	0.085	0.771
**Oxygen Inhalation treatment degree**	**8.874**	**＜0.001**

Oxyhydrogen atomizer treatment	1.597	0.207
**Noninvasive ventilator treatment**	**20.641**	**＜0.001**
Tracheal cannula treatment	2.209	0.138
Tracheotomy treatment	0.133	0.715
ECMO treatment	0.157	0.692
CRRT treatment	1.313	0.253
**ICU treatment**	**8.915**	**0.003**
Anti-infective drugs treatment	2.842	0.093
Vasoactive drug treatment	0.529	0.467
**Hormone treatment**	**34.258**	**＜0.001**

Note: The F value is not chi-square value, because we studied the antibody titers that is the numerical data is not the categorical data.

（三）Laboratory test characteristics (continuous variables)

Variables	Correlation coefficient r
**Temperature(℃)**	**0.210****
Respiration(times/min)	0.095
Pulse(times/min)	0.071
Systolic blood pressure (mmHg)	0.058
Diastolic blood pressure (mmHg)	0.036
Mean arterial pressure (mmHg)	0.046
White blood cell count ($\times 10^9$ /L)	0.060
**Neutrophil count ($\times 10^9$ /L)**	**0.184****
**Lymphocyte count (×109 /L)**	**-0.195****
Aspartate aminotransferase (U/ L)	0.028

Note: (a) *Correlation significant at the 0.05 level; **correlation significant at the 0.01. (b) Level bold lettering denotes statistically significant associations.
